# The coffee bean transcriptome explains the accumulation of the major bean components through ripening

**DOI:** 10.1038/s41598-018-29842-4

**Published:** 2018-07-30

**Authors:** Bing Cheng, Agnelo Furtado, Robert J. Henry

**Affiliations:** Queensland Alliance for Agriculture and Food Innovation, The University of Queensland, Brisbane, QLD 4072 Australia

## Abstract

The composition of the maturing coffee bean determines the processing performance and ultimate quality of the coffee produced from the bean. Analysis of differences in gene expression during bean maturation may explain the basis of genetic and environmental variation in coffee quality. The transcriptome of the coffee bean was analyzed at three stages of development, immature (green), intermediate (yellow) and mature (red). A total of more than 120 million 150 bp paired-end reads were collected by sequencing of transcripts of triplicate samples at each developmental stage. A greater number of transcripts were expressed at the yellow stage. As the beans matured the types of highly expressed transcripts changed from transcripts predominantly associated with galactomannan, triacylglycerol (TAG), TAG lipase, 11 S and 7S-like storage protein and Fasciclin-like arabinogalactan protein 17 (FLA17) in green beans to transcripts related to FLA1 at the yellow stage and TAG storage lipase SDP1, and SDP1-like in red beans. This study provides a genomic resource that can be used to investigate the impact of environment and genotype on the bean transcriptome and develop coffee varieties and production systems that are better adapted to deliver quality coffee despite climate variations.

## Introduction

Arabica coffee (around 70% of world coffee consumed), is one of the most important and valuable commodities traded internationally^1^. The coffee bean is a tropical dicotyledonous albuminous seed, with a copious endosperm and a tiny embryo. Generally, seeds have evolved intricate strategies to reserve nutrients and take advantage of the pericarp and pulp to encourage dispersal by animals^2^. The composition of the coffee bean determines the quality of the coffee. An improved understanding of the molecular basis of determination of bean composition is required to support enhanced coffee production and genetic improvement especially in response to climate change.

Numerous biochemical studies have been conducted on the genetic and environment factors influencing the accumulation of key components of the bean^3,4^. Genetic control of these processes can be investigated by the study of changes in gene expression through bean ripening, including transcripts regulating bean filling as well as in response to stress^2,5,6^. Early studies applied RT-PCR (coffee beans) or microarrays to mainly coffee leaves or seedlings^5,7,8^. More recently, different tissues of Arabica coffee including flowers, leaves and fruit pericarp have been subjected to transcriptome analysis. A genome wide study recently reported identification of key genes regulating the lipid and diterpene contents of Arabica coffee bean^4^. However, analysis of the genetics of other essential components of the bean contributing to coffee quality was not included in these studies^9^. Importantly, the absence of a reference genome or transcriptome further limited previous studies. Additionally, gaps remain in knowledge of gene interactions and how expression varies at the global scale at specific ripening stages and finally determines the quality and regeneration capability of the bean.

In this study, different development stages, green, yellow and red coffee beans (other than exocarp or mesocarp) were collected and the RNA was sequenced for transcriptome analysis. A recent long read sequencing full-length (LRS) coffee bean transcriptome was used as a reference to facilitate transcriptome analysis in the ripening coffee bean^10^.

The aim was to understand the progress of accumulation of the key component in the coffee bean through ripening and the molecular basis of genetic regulation of coffee quality and deliver a platform for the study of genotypic and environmental influences on the coffee transcriptome and coffee quality.

## Results

**Figure 1:**
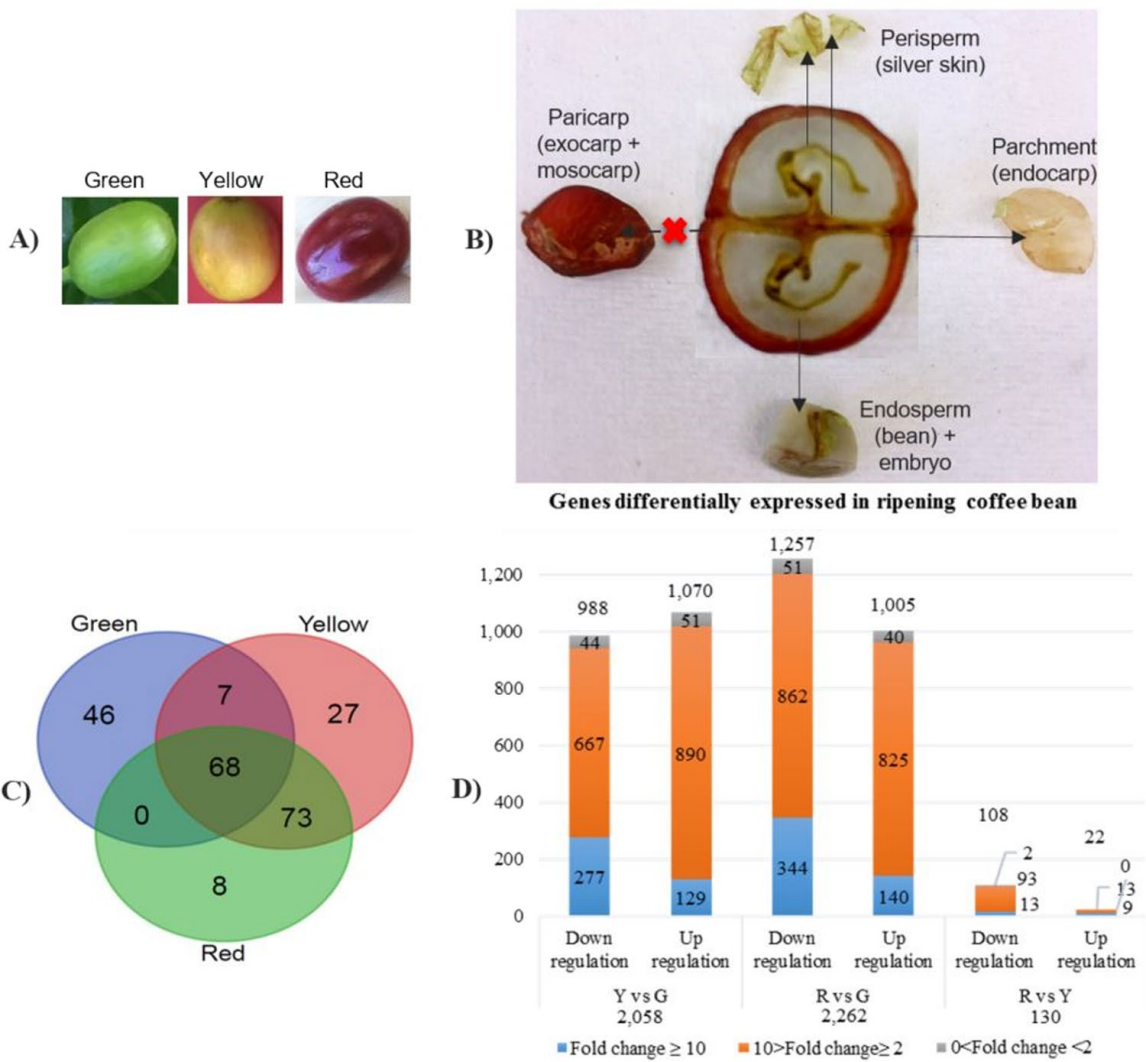
Overview of ripening coffee bean transcriptome. (**a**) Coffee cherries of different development stages. (**b**) Tissue representatives of coffee beans used in this study (pericarp was discarded). (**c**) Venn graph of highly expressed genes (TPM > 500) in green, yellow and red coffee beans. (**d**) The distribution of differentially expressed genes (up/down regulations) of ripening coffee beans according to fold change (FDR p-value ≤ 0.01, TPM (max group means) >10).

Nine coffee bean transcriptome datasets were generated in this study, including green, yellow and red stages, all in triplicate (Fig. 1a). As pericarp (including exocarp and mesocarp) was removed before RNA extraction, the coffee bean in this study refers to endocarp, perisperm (seed coat), endosperm and embryo (encapsulated by endosperm) (Fig. 1b).

### Raw read processing

A total of 120,784,786 reads were generated from the nine coffee bean transcriptome datasets (Supporting Information Table S1). This number was slightly reduced (117,539,360) after trimming. For individual samples, the number of trimmed reads were between 4,602,943 and 23,905,608. A range of 48.37% and 64.05% of reads were mapped to the long-read sequencing coffee bean transcriptome (coffee LRS transcriptome).

The yellow stage has the highest number of genes expressed (43,552 transcripts, TPM > 1), compared to red and green stages (43,257 and 36,388 transcripts) (Supporting Information Fig. S1). Functional annotation of the expressed transcripts revealed that GO terms associated with the metabolic process, catalytic activity and cell part, ranked as the most abundant in “biological process”, “molecular function” and “cellular components” respectively (Supporting Information 1 Fig. S2). The lower number of transcript expression was observed at green stage.

The top three pathways with the most transcripts expressed were purine pathway, thiamine metabolism, etc (Supporting Information 1 Table S2). Starch and sucrose metabolism and phenylpropanoid biosynthesis were the sixth and ninth most enriched pathway. The top 30 pathways with the highest number of transcripts expressed were from 11 parent-pathway groups. The dominant parent-pathway group was carbohydrate metabolism, enriched with the highest number of pathways (seven pathways), including starch and sucrose metabolism and galactose metabolism. This is followed by the amino acid and lipid metabolism parent-group, which relating to five and three metabolism pathways, individually.

### Highly expressed transcripts through coffee bean ripening

#### Overview

Of all the HEGs, more unique transcripts were expressed over TPM500 at the green stage (46 transcripts) other than yellow and red stages (27 and 8) (Fig. 1c). A total of 68 common transcripts were expressed at all the three development stages. A significantly higher number of common transcripts (73) were expressed at both the yellow and red stages, while the number of common transcripts (seven) expressed at the green and the red stages were the same as that of the first two stages.

#### Intensive lipids formation at the green stage

The top ten HEGs in green, yellow and red coffee beans were extracted individually for further analysis (Table 1). Other than three unnamed protein products, a ncRNA was one the most abundant transcript at all stages, peaking at the green stage and decreasing at the last two stages. Two non-specific lipid transfer (LTP) A-like transcripts, showed extremely high expression in green coffee beans (33,227 and 18,563) but decreased dramatically in yellow stage (5,488 and 1,446) until maturity (1,260, 378 and 1,165). This suggested a high level of lipids may accumulate and transported from this stage. A similar drop was also identified in alpha-galactosidase 2, from 10,120 in the green stage to more than ten times lower in yellow stage (830) and red stage (351). Fewer changes were characterized in transcripts encoding 11S storage globulin (bean storage protein) and metallothionein type 3 (important for bean development), presenting maximum expression in green coffee beans (9,994 and 7,709 respectively), while gradually dropped until red coffee beans (4,059 and 6,829 respectively). Transcripts encoding for kirola-like protein and dehydrin DH1a (response to desiccation), peaked at the yellow stage and decreased slightly in red stage.

**Table 1:** Top ten highly expressing transcripts in green, yellow and red stages of coffee beans (TPM).

Top10 in green	Green	Yellow	Red	Sequence Description
**c80141/f1p1/450**	**94,493**	15,075	22,440	Cucumis melo uncharacterized LOC107990804 (LOC107990804) transcript variant ncRNA
c63112/f22p4/653	**33,227**	5,488	1,260	non-specific lipid-transfer A-like
**c31970/f1p3/511**	**30,270**	26,070	29,246	hypothetical protein COLO4_02306
c24248/f1p0/1181	**29,834**	3,567	4,370	Sesamum indicum NEDD8-specific protease 1 (LOC105170338) transcript variant mRNA
**c34759/f1p0/1129**	**25,585**	17,528	16,531	unnamed protein product
c8287/f57p5/686	**18,563**	1,446	378	non-specific lipid-transfer A-like
c152969/f1p43/1468	**10,120**	830	351	alpha galactosidase 2
c153711/f216p83/1761	**9,994**	7,687	4,059	11S storage globulin
c13322/f1p0/738	10,004	7,731	8,460	Nicotiana sylvestris uncharacterized LOC104249947 (LOC104249947) transcript variant ncRNA
c73790/f1p0/2722	15,260	4,001	5,532	pyrophosphate–fructose 6-phosphate 1-phosphotransferase subunit beta
Top10 in **Yellow**	Green	**Yellow**	Red	Sequence Description
**c31970/f1p3/511**	30,270	**26,070**	29,246	hypothetical protein COLO4_02306
**c34759/f1p0/1129**	25,585	**17,528**	16,531	unnamed protein product
**c80141/f1p1/450**	94,493	**15,075**	22,440	Cucumis melo uncharacterized LOC107990804 (LOC107990804) transcript variant ncRNA
*c35373/f1p2/1001*	4,961	**10,731**	11,805	PSII 32 kDa (mitochondrion)
c153711/f216p83/1761	9,994	**7,687**	4,059	11S storage globulin
*c56830/f3p1/506*	7,709	**7,576**	6,829	metallothionein type 3
*c63165/f2p6/845*	1,759	**6,977**	7,010	unnamed protein product
*c31995/f48p5/939*	1,783	**6,744**	5,988	dehydrin DH1a
*c42276/f1p3/825*	2,200	**6,278**	7,029	unnamed protein product
*c153289/f6p3/751*	1,930	**5,841**	5,180	kirola-like
Top10 in **Red**	Green	Yellow	**Red**	Sequence Description
**c31970/f1p3/511**	30,270	26,070	**29,246**	hypothetical protein COLO4_02306
**c80141/f1p1/450**	94,493	15,075	**22,440**	Cucumis melo uncharacterized LOC107990804 (LOC107990804) transcript variant ncRNA
**c34759/f1p0/1129**	25,585	17,528	**16,531**	unnamed protein product
*c35373/f1p2/1001*	4,961	10,731	**11,805**	PSII 32 kDa (mitochondrion)
*c42276/f1p3/825*	2,200	6,278	**7,029**	unnamed protein product
*c63165/f2p6/845*	1,759	6,977	**7,010**	unnamed protein product
*c56830/f3p1/506*	7,709	7,576	**6,829**	metallothionein type 3
*c31995/f48p5/939*	1,783	6,744	**5,988**	dehydrin DH1a
*c153289/f6p3/751*	1,930	5,841	**5,180**	kirola-like
c24248/f1p0/1181	56,405	14,965	**18,132**	Sesamum indicum NEDD8-specific protease 1 (LOC105170338) transcript variant mRNA

Transcripts in the top-ten list but common between the green and yellow stages and between yellow and red stages are indicated in underline and italic text respectively.

#### More changes in the comparison of the red vs green stage

Three comparisons between developmental stages were conducted in this study of the ripening Arabica coffee bean transcriptome, yellow vs green (Y vs G), red vs green (R vs G) and red vs yellow (R vs Y) included. The highest number of differentially expressed transcripts (DEGs) were shown in the R vs G comparison (2,262), including the most downregulated DEGs (1,257) (Fig. 1d). Only 130 DEGs were seen in R vs Y, including 108 downregulated genes and 22 upregulated genes. A total of 2,058 DEGs were characterized in Y vs G, with the most up-regulated DEGs (1,070). The majority of DEGs were distributed within the range of two to ten times fold change, while only a few varied less than two times fold change.

The top ten up and down-regulated DEGs from individual comparisons were extracted in Table 2 to understand the most significant changes in coffee bean ripening. These transcripts included pathogen relevant, cell function and key chemical biosynthesis transcripts.

**Table 2:** Expression (TPM) of top ten down/upregulated differentially expressed genes in the comparison of yellow vs green, red vs green and red vs yellow.

Comparisons	DOWN regulations (Sequence ID)	log_2_ fold change	FDR p-value	Expression values			Sequence description
				Green	Yellow	Red	
Yellow vs Green	c59581/f2p4/1062	−11.44	0.00E + 00	199.16	0.07	0.36	12658Cc06_g14410
	c32395/f2p4/1179	−10.38	0.00E + 00	90.46	0.19	0.00	Class III chitinase (chi2)
	c12812/f5p0/577	−9.85	0.00E + 00	2550.72	5.81	4.67	unnamed protein product
	c51638/f2p4/1139	−9.52	0.00E + 00	50.59	0.11	0.10	Class III chitinase (chi2)
	c76145/f1p1/642	−9.02	0.00E + 00	2878.44	10.98	10.79	ubiquitin 40 S protein S27a
	c1950/f3p0/1246	−8.57	0.00E + 00	81.57	0.40	0.68	GDSL esterase lipase APG-like
	c29520/f6p2/1511	−8.40	0.00E + 00	78.92	0.30	0.77	O-fucosyltransferase family
	c6862/f2p2/1505	−8.34	0.00E + 00	29.29	0.30	0.25	GDP-fucose O-fucosyltransferase
	c55541/f6p4/1596	−8.24	0.00E + 00	49.02	0.35	0.65	WAT1-related At2g37460-like
	c67536/f1p2/961	−8.22	0.00E + 00	5278.44	34.48	25.48	13999Contig1271
Red vs Green	c227/f23p5/1246	−10.39	0.00E + 00	231.93	8.32	0.37	hyoscyamine 6-dioxygenase-like
	c59581/f2p4/1062	−10.15	0.00E + 00	199.16	0.07	0.36	12658Cc06_g14410
	c12812/f5p0/577	−10.11	0.00E + 00	2,550.72	5.81	4.67	unnamed protein product
	c47253/f1p1/1271	−9.21	0.00E + 00	209.43	6.9	0.55	hyoscyamine 6 beta-hydroxylase
	c76145/f1p1/642	−9	0.00E + 00	2,878.44	10.98	10.79	ubiquitin 40 S protein S27a
	c30689/f6p3/1067	−8.85	0.00E + 00	153.84	2.49	0.79	12658Cc06_g14410
	c154384/f5p2/662	−8.82	0.00E + 00	221.23	2.77	0.7	vacuolar invertase inhibitor
	c11158/f3p5/1395	−8.81	0.00E + 00	85.17	0.86	0.33	dehydration-responsive element-binding 2D-like
	c67536/f1p2/961	−8.69	0.00E + 00	5,278.44	34.48	25.48	13999Contig1271
	c44011/f13p4/584	−8.38	0.00E + 00	4,673.02	138.76	26.39	unnamed protein product
Red vs Yellow	c9963/f1p1/900	−4.4	0.00E + 00	790.69	309.83	15.03	E3 ubiquitin- ligase SHPRH isoform X1
	c2057/f9p6/929	−4.04	0.00E + 00	1412.25	537.78	33.22	luminal-binding 5-like
	c72328/f2p11/3062	−4.02	0.00E + 00	137.62	37.07	2.29	electron transfer flavo subunit beta, mitochondrial
	c8307/f2p1/1218	−2.88	2.13E − 12	511.49	107.26	14.63	ADP-ribosylation factor 1-like
	c424/f42p13/1416	−2.47	2.13E − 12	208.87	132.87	24.38	endonuclease V
	c46628/f1p11/1830	−3.01	7.11E − 12	115.19	24.31	2.46	MYB transcription factor MYB90
	c32622/f2p2/3386	−2.35	1.07E − 11	6.48	48.55	9.85	mannose-1-phosphate guanyltransferase alpha
	c23452/f5p4/795	−2.34	1.66E − 11	228.91	122.02	25.05	cinnamoyl- reductase 2-like
	c3523/f1p2/916	−1.98	3.73E − 11	145.87	217.53	56.09	unnamed protein product
	c2393/f4p5/950	−3.89	1.02E − 10	539.03	201.10	14.12	endonuclease V isoform X1
**Comparisons**	**UP regulations (Sequence ID)**	**Log2 fold change**	**FDR p-value**	**Expression values**			**Sequence description**
				**Green**	**Yellow**	**Red**	
Yellow vs Green	c70460/f8p4/1626	8.41	0.00E + 00	0.78	406.11	996.73	trans-resveratrol di-O-methyltransferase-like
	c14624/f5p2/1077	6.27	0.00E + 00	2.60	354.84	221.06	germin subfamily T member 2
	c5092/f1p2/1158	6.20	0.00E + 00	0.97	108.21	295.30	MYB transcription factor
	c87219/f6p8/5061	6.14	0.00E + 00	0.19	28.59	61.20	retrotransposon Ty1-copia subclass
	c21121/f1p0/941	6.11	0.00E + 00	0.57	66.88	44.72	germin 2-1
	c101202/f2p0/662	5.97	0.00E + 00	0.58	76.30	69.50	pathogenesis-related 1
	c32281/f2p1/2368	5.92	0.00E + 00	0.60	55.18	41.06	probable rhamnogalacturonate lyase B
	*c128223/f1p1/2236*	5.63	0.00E + 00	0.44	47.61	129.42	MYB transcription factor MYB90
	c41502/f4p9/1331	5.62	0.00E + 00	4.65	453.03	411.65	probable xyloglucan endotransglucosylase hydrolase B
	c66442/f1p1/1028	5.49	0.00E + 00	1.96	137.11	115.80	pectinesterase inhibitor 11
Red vs Green	c70460/f8p4/1626	9.68	0.00E + 00	0.78	406.11	996.7	trans-resveratrol di-O-methyltransferase-like
	c5092/f1p2/1158	7.61	0.00E + 00	0.97	108.21	295.3	MYB transcription factor MYB90
	c87219/f6p8/5061	7.23	0.00E + 00	0.19	28.59	61.2	retrotransposon Ty1-copia subclass
	*c128223/f1p1/2236*	7.02	0.00E + 00	0.44	47.61	129.4	MYB transcription factor MYB90
	c122295/f1p5/5026	6.56	0.00E + 00	0.47	41.21	105.4	retrotransposon Ty1-copia subclass
	c88329/f1p1/2084	6.31	0.00E + 00	0.2	13.33	27.42	MYB transcription factor MYB90
	c63916/f1p0/1089	6.18	0.00E + 00	0.24	20.96	49.39	beta-glucosidase 44-like
	c10950/f7p1/863	5.92	0.00E + 00	0.53	43.84	97.24	lipid transfer
	c52265/f1p6/1138	5.82	0.00E + 00	6.09	405.31	686	MYB transcription factor MYB90
	c101202/f2p0/662	5.8	0.00E + 00	0.58	76.3	69.5	pathogenesis-related 1
Red vs Yellow	c21728/f1p0/1557	4.19	2.84E − 10	1.30	3.02	59.72	luminal-binding 5-like
	c13553/f10p5/1234	5.09	5.26E − 09	17.61	0.68	25.13	electron transfer flavo subunit mitochondrial
	c28681/f1p0/995	4.75	5.26E − 09	2.54	0.94	30.53	ADP-ribosylation factor 1-like
	*c128223/f1p1/2236*	1.39	3.27E − 06	0.44	47.61	129.42	MYB transcription factor MYB90
	c78857/f1p1/1552	1.36	3.56E − 06	7.22	39.18	103.33	cinnamoyl- reductase 2-like
	c89078/f1p2/5144	4.5	2.47E − 05	18.17	1.74	37.43	endonuclease V isoform X1
	c113302/f1p3/1573	1.40	0.00015	3.32	46.00	123.83	cinnamoyl- reductase 2-like
	c67038/f1p5/2024	1.74	0.00022	0.81	12.82	43.78	ECERIFERUM 1-like
	c22721/f1p0/827	3.23	0.00023	1.59	6.99	70.51	luminal-binding 5-like
	c53207/f3p2/1601	1.21	0.00086	3.81	34.66	81.76	cinnamoyl- reductase 2-like

Words in italic means common in all comparisons, while bold words indicate common in either two stages.

In the comparison of Y vs G, two class III chitinase (chi2) transcripts were highly expressed in green coffee beans and were absent (TPM < 1) in yellow stage. Similarly, a dramatic decrease was shown in a lipid degradation transcript (controls GDSL esterase lipase APG-like protein), an O-fucosyltransferase family transcript (relating to mannan biosynthesis and galactomannan accumulation), an amino acid transport transcript (*WAT1-related protein-like*), and a cellular function transcript (regulating ubiquitin 40S protein S27a). The ubiquitin 40S protein S27a related transcript also demonstrated a significant change in the comparison of R vs G. Additionally, a significant decrease from green to red coffee beans was characterized in a cell wall vascular inhibitor of fructosidase 1-like (*vacuole invertase inhibitor*). In the comparison of R vs Y, E3 ubiquitin- ligase SHPRH isoform X1, Luminal-binding 5-like, cinnamoyl- reductase 2-like etc. degraded in red stage.

Numerous MYB transcription factors (*MYB90)* were identified as upregulated DEGs in top ten DEGs of all comparisons, increasing from green to maturity (maximum expression was TPM: 686). A trans-resveratrol di-O-methyltransferase-like transcript, catalyzing pterostilbene (antifungal and pharmacological function) biosynthesis and a transposon elements variant gene (*retrotransposon Ty1-copia subclass*) were upregulated in both comparison of Y vs G and R vs G as they increased sharply from green to red stage. From the green stage, cell wall degradation DEGs, such as probable rhamnogalacturonate lyase B (probably pectin degradation) and probable xyloglucan endotransglucosylase hydrolase B (*XTHB*, cleaving primary cell wall xyloglucan polymers and contributing to the construction of the growing tissues) were upregulated at the yellow stage. Meanwhile, transcript expression of the pectinesterase inhibitor 11 transcript, maintaining the integrity of cell walls, was increased and peaked at the yellow stage. Steady growth was detected in beta-glucosidase 44-like, lipid transfer and pathogenesis-related 1 transcripts. Moreover, the comparison of R vs Y included cell function transcripts, like *Luminal-binding* 5*-like*, *endonuclease V* and three transcripts probably related to phenolic compounds, *cinnamoyl- reductase 2-like*.

### Storage compounds accumulated through bean maturity

**Figure 2:**
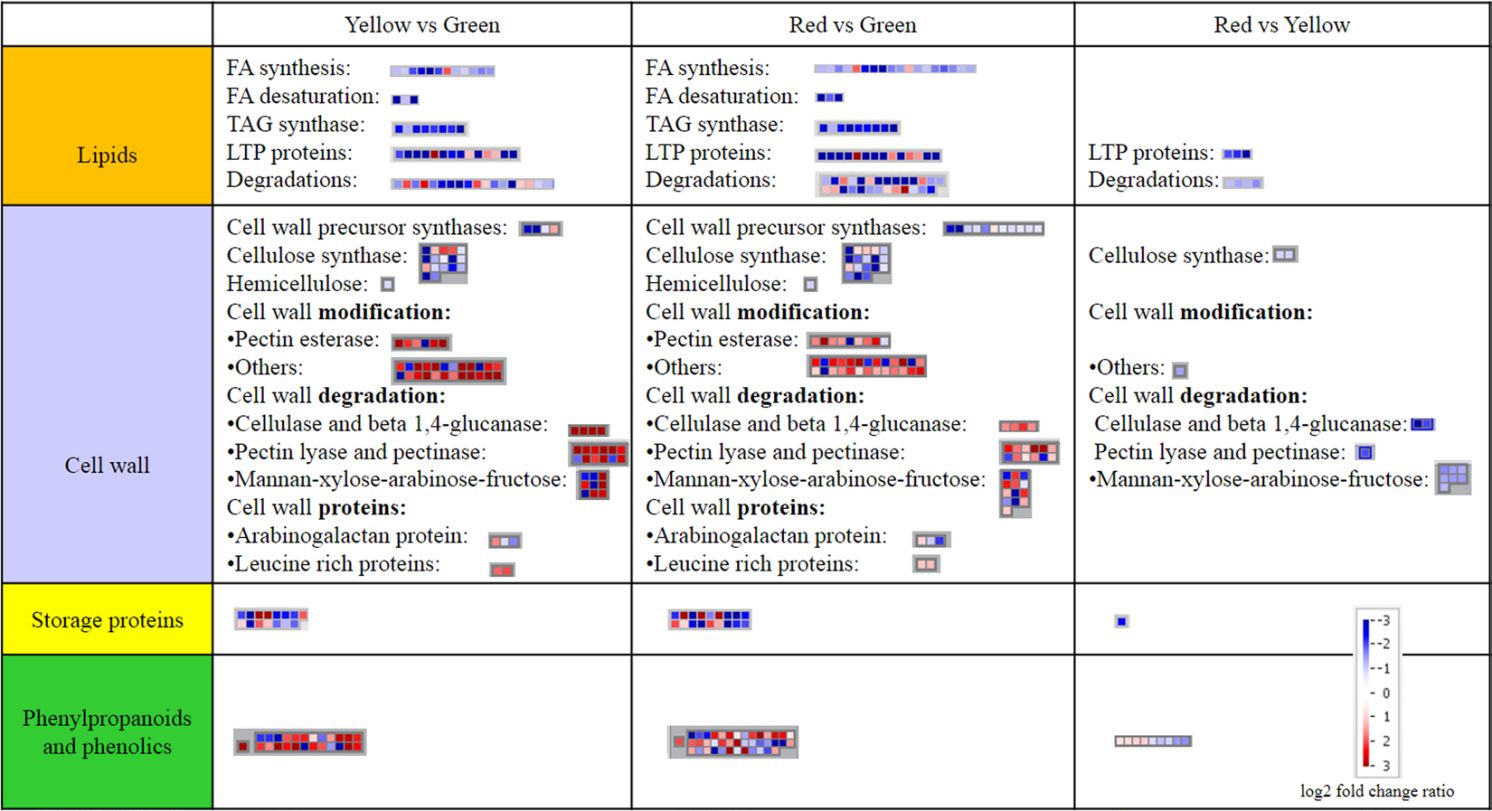
Candidate genes involved in the key storage components of the coffee bean differentially expressed through ripening. Up and down regulations were shown in red and blue boxes. Darker color indicates larger fold change (log2 transformed fold change). FA, fatty acids; TAG, triacylglycerol; LTP, lipid transfer protein.

An association study of the key storage component related DEGs was conducted with lipid, cell wall component, storage protein and phenylpropanoid related DEGs in ripening coffee bean (Fig. 2). Major changes were observed in the comparison of Y vs G and R vs G. More DEGs were assigned in the comparison of R vs G, while only a few shown in a comparison of the last two stages (R vs Y).

#### Major lipids accumulation at the green stage

The main lipid-related DEGs went through a decrease in expression compared to levels in green coffee beans, especially fatty acid desaturation (omega-6-desaturase, critical for biosynthesis of linoleic acid), TAG synthase (oil body oleosin family proteins) and lipase (for TAG degradation) (Supporting Dataset). One omega-6-desaturase declined dramatically (log2 ratio: −6.10) at the yellow stage in comparison with the green stage (Supporting Dataset). Similarly, non-specific LTP protein decreased sharply (maximum log2 ratio: −8.16 in R vs G) compared to the green stage, supporting the idea of lipids predominantly accumulating in green beans. Only a limited number of DEGs remained in the comparison of the last two stages, and they were all down-regulated. This includes three non-specific lipid transfer proteins (phospholipid transfer protein) and four GDSL-like Lipases. Therefore, TAG is likely to be synthesized in green beans, which accompanies its degradation by lipase. Linoleic acid was also apparently formed in green bean.

#### The flow of cell wall components

Most down-regulated DEGs in the comparison of Y vs G were found to relate to cell wall precursor synthase, hemicellulose synthase (only downregulation), cellulose synthase and arabinogalactan protein (AGPs) DEGs (Supporting Dataset). Only one UDP-XYL synthase (*USX*, UDP-D-xylose biosynthesis) transcript 2-like was upregulated in Y vs G and R vs G in cell wall precursor DEGs. The others were downregulated either in Y vs G or R vs G. Mannose-1-phosphate guanylyltransferase (*CYT*, involved in GDP-mannose biosynthesis) was downregulated in the last two stages. Downregulations include nucleotide-rhamnose synthase/epimerase-reductase, *USX6* and *USX6-like*, UDP-glucose 6-dehydrogenase 5 (provides nucleotide sugars for cell-wall polymers).

Decreased cellulose synthase DEGs are transcript 1 and 2 (*CESA1, 2*), COBRA-like protein 1, *COBRA-like* and CESA-like transcript (mannan synthase 1-*MS1*, which peaks in the green stage), while upregulation was seen in two CESA-like transcripts with upregulation observed in *MS2* (peaking at the yellow stage). AGP related DEGs were downregulated in *FLA17* and upregulated as was seen in *FLA1* (top expression in yellow stage). Hemicellulose related DEG, regulating hydroxyproline O-galactosyltransferase 6 (translocates galactose from UDP-galactose to the residues of AGPs), declined in the yellow and red stages compared to the green stage. However, the only increases in transcript expression were shown in the comparison of Y vs G and R vs G in *cellulase* (*CEL3* and 5) and *beta 1,4-glucanase* (such as transcript 6), with a peak expression in the yellow stage. Leucine-rich protein (LRR) associated DEGs were also seen to rise in Y vs G and R vs G. A large number of DEGs were found in pectin degradation (pectin esterases, lyses and pectinases), which were mainly upregulated in Y vs G and R vs G. A major rise of transcript expression was also observed in other cell wall modification DEGs including numerous expansin (and expansin-like) transcripts (isoform 6, 8, 10, 11 and 15) and XTH transcripts (isoform B, 2, 6, 12, 23, 30). Expression of *BGAL-like*, probable beta-D-xylosidase 7 and *mannan endo-1,4-beta-mannosidase 5-like* (one transcript variant was upregulated) DEGs declined at the yellow stage. Similar patterns continued in the second comparison, R vs G. A higher number of DEGs were associated with cell wall precursor synthases, cellulose synthases, cell wall modifications and mannan degradation. However, in the last comparison, R vs Y, all DEGs were downregulated. Altogether, cellular precursors, hemicellulose, *FLA17*, *CESA1* and *CESA2* and *MS1* increased relative to the highest expression at the green stage. Peak expression shifted to cellular degradation (*CEL3* and *5*) and *MS2* at the yellow stage and pectin degradation and LRR in the last two stages.

#### Major storage protein accumulation at the green stage

In terms of storage protein related DEGs, both up and down regulations were identified in both Y vs G and R vs G comparisons, while no DEGs were assigned in the comparison of R vs Y (Supporting Dataset). Altogether, significant decreases were identified in eight storage protein related DEGs in the yellow and red stage compared to green coffee beans. They were four 11 s globulin (*11S*), three 7S-like globulin transcripts (*7S-like*) and glutelin type A2 (*GLUA2*) DEGs. In addition to these eight DEGs, one more *11S* and a patatin (*Pat*) 2 also decreased in the red stage in contrast to green beans. In contrast, upregulation was observed in three *Pat6*, *GLUA3*, TAG lipase *SDP1*, *SDP1-like* (storage lipids degradation in bean germination) and *SDP1-like* DEGs in the comparisons of Y vs G and R vs G (larger fold change at the red stage). Transcripts regulating these proteins are likely to peak at maturity. One more *GLU*, type B5 (*GLUB5*), was also more highly expressed at the red stage rather than in green coffee beans. Hence, *11S*, *7S-Like* and *GLUA2* storage probably occurred since the green stage, while *SDP1*, *SDP1-like*, GLUA3, *Pat6*, accumulated at the end.

#### Phenylpropanoids

Most CGAs related transcripts peaked at the yellow stage, with a significant increase since the green stage and decreased dramatically in the red stage (FDR corrected p-value < 0.001). Exceptions were phenylalanine ammonia-lyase (*PAL*) 4 and 4-coumarate CoA ligase (*4CL*) 7, which increased from green coffee beans till maturity. This suggested that CGAs were likely to be mainly formed from the yellow stage and *4CL7* probably contributes to further accumulation of CGAs or lignin in later stages.

### Co-expression network of the bean storage genes

**Figure 3:**
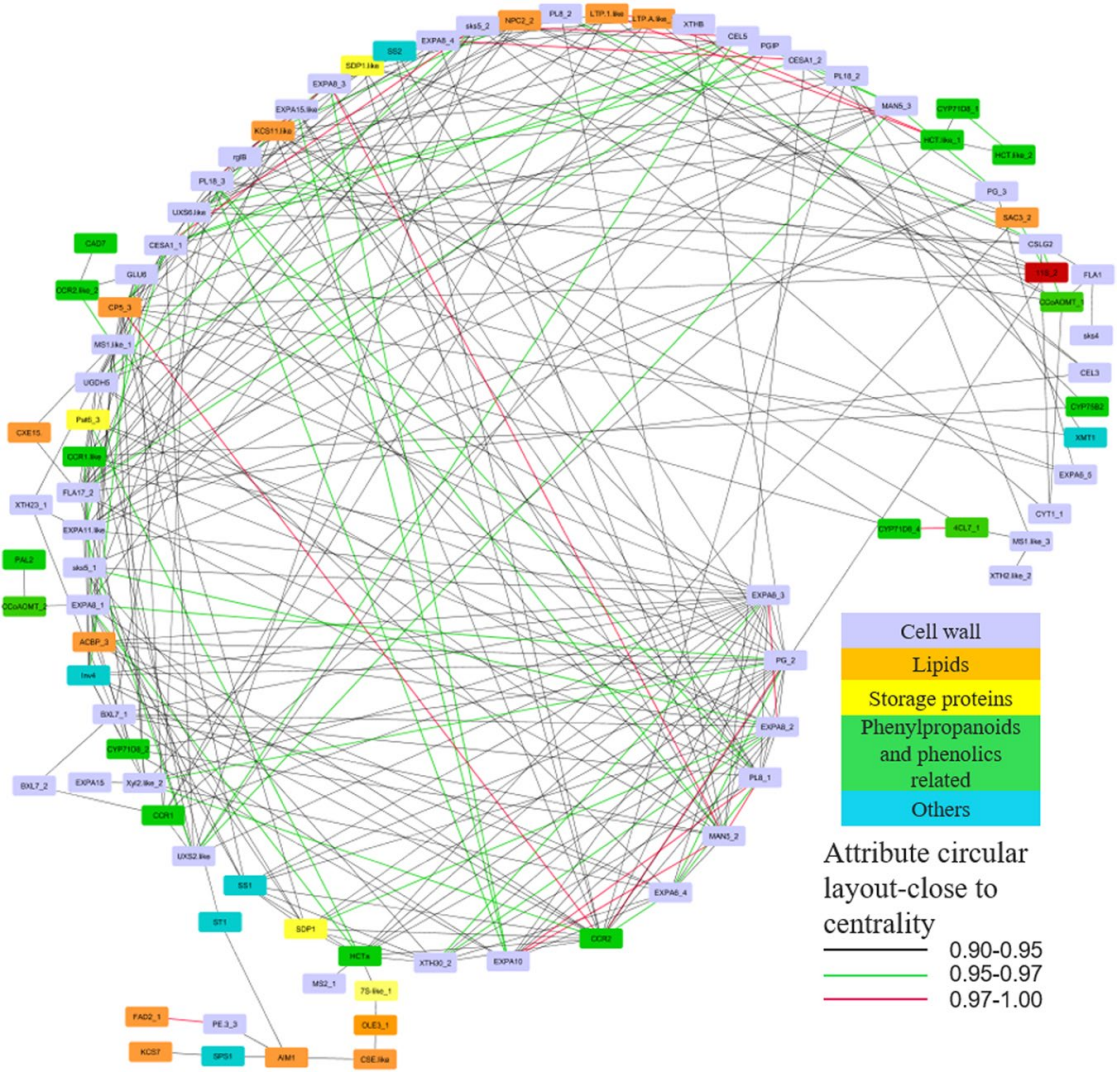
Co-expression network of key storage genes according to weight. Each triangular indicates a candidate gene. Black, green and red lines indicating weight ranged in 0.90–0.95, 0.95–0.97 and 0.97–1.00.

Four groups of transcripts were targeted for co-expression network analysis, including lipid, cell wall, storage protein and phenylpropanoids DEGs (Fig. 3). The aim was to investigate the connections among key bean storage/quality attributed transcripts. A great number of the cell wall and phenylpropanoids DEGs were filtered out in the co-expression module (weight ≥0.9), followed by lipid, other metabolites, and storage protein related transcripts.

Different transcripts of alpha-expansin transcripts (*EXPA*) played a key role in this network module, especially *EXPA6_3* (*EXPA6_3* transcript variant 3). *EXPA6_3* was co-expressed with 22 transcripts from all five categories, mainly cell wall-related. The top five connected transcripts to *EXPA6_3* were *EXPA* 8_2 (0.9709), probable xyloglucan endotransglucosylase hydrolase 30 (*XTH30_2*, 0.9681), probable pectin lyase 8 (*PL8_1*, 0.9402), polygalacturonase-like (*PG_2*), *EXPA11-like* (0.9399), etc. Another core transcript connected with all five transcript groups was *PG_2*. *PG_2*, identified as being connected to cinnamoyl- reductase 2 (*CCR2*), beta-xylosidase/alpha-L-arabinofuranosidase 2-like (*Xyl2-like_2*) and shikimate O-hydroxycinnamoyltransferase transcript a (*HCTa*). This suggested *PG_2* is essential in cell wall modification and probably interacts with phenylpropanoid and phenolic biosynthesis.

A lipid DEG, *CP5_3*, functions as a membrane related protein and was one of the pivotal transcripts in the co-expression network. The top five co-expression were from different groups, comprise of *CCR2*, *EXPA8_1*, *PG_2*, *11S_2*, etc. In addition, 3-ketoacyl-CoA synthase 11-like (*KCS11-like*, biosynthesis of very long chain fatty acids), unanimously expressed with transcripts from all categories, was another central transcript from the module. The co-expressed transcripts were *CP5_3*, non-specific phospholipase C2, cytochrome P450 71D8, EXPA8_1, *CCR2*, etc. The relationship of these diverse transcripts suggested that *CP5_3* and *KCS11-like* are essential in bean ripening and nutrient reservation.

*CCR2* (biosynthesis of phenolics), was among the most important transcripts from the network. Other than the above connections, *CCR2* was also associated with diverse categories with the highest impact on *EXPA6_4*, *Xyl2-like_2*, and *EXPA8_1*. Hence, it is likely *CCR2* is pivotal to cell wall expansion and modification. The next important phenylpropanoids related DEGs was *HCTa*, correlating to *EXPA8_1*, *Pat6_3*, *MS2_1*, and *7S-like_1*, indicating a key role in cell wall expansion and storage protein biosynthesis.

Very few storage protein related transcripts were distributed in this co-expression network module and they were co-expressed with a lower number of transcripts. Among them, *SDP1* and *11S* were relatively important. *SDP1* expression was also found to be concurrent with *SKU5* similar 5 (*sks5_1*), *XTH30_2*, *CCR2* and *HCTa*, while 11S correlated with phosphoinositide phosphatase SAC3 (*SAC3_1*), *CCR2*, *SDP1-like*, and *KCS11-like*.

## Discussion

As a storage tissue, nutrients are formed and stored through complicated pathways in beans to support the embryo development and reproduction of the plant. Arabica coffee beans are composed of cell wall polysaccharides (CWP, 50% of the dry mass), lipids (13–17%), proteins (11–15%), sucrose (7–11%), and CGAs (5–8%)^3,4,11^. The major CWP in the young coffee bean, comprise arabinogalactan (~50%), cellulose, pectic polymers (~20%) and galactomannans (10%)^5,11,12^. However, at maturity, this has changed to arabinogalactan-proteins (~30%, AGPs), cellulose (15%), pectic polymers (~5%) and galactomannan (50%)^12,13^. Lipids were reported to be mainly composed of triacylglycerol (TAG, 70–80% of lipids), while the major storage protein was identified as 11S globulin (45%)^5,11,13^.

This study reveals the peak expression of candidate transcripts related to major storage compounds was in green beans at the initiation of the storage phase, for example, galactomanan, FLA17, TAG, linoleic acid, 11S, 7S-like and glutelin A2. The main accumulation of FLA1 started at the yellow stage. Transcripts encoding SDP1, SDP1-like, Glueteline A3, Pat2, storage proteins reached a maximum expression at the red stage. In addition, pectin was mainly degraded at the yellow and red stages. The lower number but wider range of HEGs from green coffee beans was probably associated with the initiation of the storage phase, where a large number of key components were formed predominantly at this stage.

Galactomannan is a typical storage compound for legumes (>30% of the bean dry weight), coffee, and palms^14^. Galactomannan can react with proteins through Millard reactions to produce volatile components, contributing to the coffee flavour^15^. The high viscosity of the remaining galactomannan accounts for the texture, namely “body”, of the coffee beverage^16^. For the plant itself, the low solubility and high viscosity galactomannans are a stable storage reserve that provides strong cells to prevent osmotic stress, microbial attack or mechanical damage^14,17^. In seed germination, galactomannans were degraded by α-galactosidase (encoded by *gal*), endo-β-mannanase and β-mannosidase to provide carbon and energy for embryo development^14,18^.

Golublins such as 11S and 7S are major storage proteins in legumes^19,20^. Glutelins and prolamins are the predominant proteins in cereals, such as rice (>60%)^21^. 11S globulin was found to be the major storage protein in coffee^11^. In addition, this study suggested the presence of other potential storage proteins of 7S-like, SDP1, SDP1-like, glutelinA2, A3, and Patatin 6. In contrast, the major storage proteins of the exalbuminous seed of Arabidopsis, mainly 2S and 12S protein (one-third of the total protein) were mainly formed late in bean development^22^. Decreased accumulation through ripening was also shown in the TAG and linoleic acid (fatty acids desaturation) storage in coffee and Arabidopsis seeds (stored at the late stage)^23^. Different storage pattern may result from a different structure (copious endosperm in the coffee bean) and function of bean tissues. In Arabidopsis, TAG storage lipase SDP1 and SDP1-like were found to catalyze more than 90% of the TAG in seed germination^24^. The accumulation of these two lipase at the end of maturity is likely to avoid unnecessary degradation of storage reserves, TAG.

The higher number of transcripts expressed at the yellow stage together with more DEGs compared to the green stage indicated a great shift at the yellow stage. A slight decrease in the number of transcripts expressed at the red stage as well as the small number of DEGs (mostly down-regulated) compared to the yellow stage, demonstrates bean maturity and fewer changes in coffee beans when the pericarp changed from yellow to red. This also indicates the end of bean maturity. Fewer components accumulated in the last two stages suggesting a possible shift to modification, such as cell wall degradation (pectin and cellulose degradation). Some or all of the coffee bean arabinogalactans were combined with proteins and present as AGPs^25^. This structure is typical in plant cell walls and has an important role in intercellular signaling and wound sealing (as a glue)^26^. Different transcripts of AGPs accumulated (*FLA17* at the early stage with *FLA1* in later stages) indicating various function through ripening in various tissues (endosperm, perisperm and embryo). Pectinesterase, lyase, and polygalacturonase catalyze pectin degradation, modifying cell walls through demethylesterification and depolymerization of pectin^27–30^. The decrease in pectin in the ripening coffee bean reaches a peak after the yellow coffee bean stage. This parallels the transcripts expressed in fleshy fruit, such as transcripts encoding polygalacturonase in Arabidopsis, which are highly expressed in ripe fruits (pericarp and mesocarp) as they soften^31^. In addition, dilution by the accumulation of other key compounds during bean ripening is probably another reason for the decline of pectin content.

Importantly, this study suggested expansin transcripts (especially *EXPA6_3*) were essential in bean ripening and storage. The higher number of the cell wall and phenylpropanoids related transcripts filtered with the storage DEGs module reveals the close relationship of transcripts from these two groups. Multiple alpha-expansin transcripts from the core co-expression network connected to transcripts from different transcript categories indicating their diverse functions in bean storage. Expansins loosen cell walls during plant growth and allow responses to the plant growth hormone, auxin^32^. There were four expansin subfamily members, *EXPA* (acid-induced), *EXPB* (beta-expansin), *EXPA-like* and *EXPB-like*^33^. It was proposed that wall loosening by expansins may be involved with a breakdown of non-covalent binding of cellulose microfibrils. This results in turgor driven polymer movement that may be inhibited by some polysaccharide-binding proteins^33,34^. However, the exact mechanism of expansin action remains unknown. In this study, expansin related DEGs were *EXPA* or *EXPA-like*. Consistently, core transcripts from the co-expression network, *EXPA6_3*, were highly stimulated with probable xyloglucan endotransglucosylase 30 (degradation of xyloglucan polymer). Co-expression was also shown with pectin and xylan degradation transcripts (*PL8*, *PG_2* and *Xyl2-like*), suggesting pectin and hemicellulose are potentially targeted by expansins or involved in the cell wall expansion phase. Other than cell wall-related transcripts, *CP5*, *SDP1* and *HCTa*, were concurrent with *EXPA6_3* suggesting a diverse interaction of *EXPA6_3*. When cell walls expand, they became vulnerable. This may result in the co-expression of *HCTa*, SDP1 and *CP5* (lipid transfer).

Plants cannot move; therefore, they have evolved numerous strategies to survive, mature and regeneration. As the bean grows to become a nutrition factory, other species like predators (virus or insects) are also interested in consuming the storage compounds generated. However, plants have evolved multiple strategies to protect beans from damage and recover from damage. For example, galactomannan and the lignified endocarp provide a physical barrier to prevent bean from being digested by predators but dispersed after consumption of the pulp^35^.

Different HEGs and DEGs characterize the complicated strategy evolved in the dicotyledonous albuminous coffee beans. Stress-related transcripts are more highly expressed in the green coffee beans, which have the protection of a thick and firm lignified pericarp. However, to protect against pathogens such as fungi, specific transcripts were expressed in green coffee beans. This was supported by the peak expression of chi2, galactomannan at this stage. The gradually hardening of endosperm then takes over the protection of the embryo and endosperm. Assorted transcripts were expressed at the yellow stage in coffee beans. As the coffee pericarp became soft and its color changed to be distinguished at the yellow stage. The brighter color (red) of the coffee pericarp attracts animals to consume the pulp but to disperse intact beans it requires more stress-responsive transcripts, which were evidenced in this research. This coincidence with digressive metabolite biosynthesis upon maturity and fewer growth transcripts. The beta-glucosidase 44-like transcript is a typical example of a plant stress resistance gene, expressed at maximum level in the ripe coffee beans. It was highly expressed in the Arabica coffee bean to activate chemical defense compounds once the cell is being attacked^36,37^. Another representative case is *actin-7*, highly expressed in red bean, related to growth and required for callus formation and response to wounding^38^.

**Figure 4:**
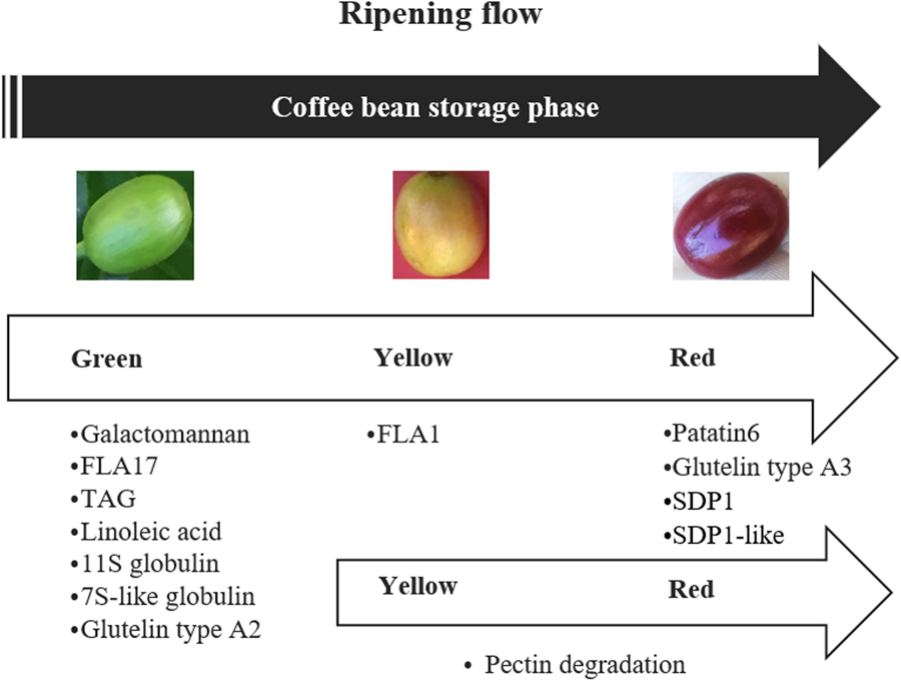
Summary of expression pattern in storage-related genes through coffee bean ripening. FLA, faccilin-like arabinogalactan; TAG, Triacylglycerol, SDP1, TAG storage lipase SDP1.

In conclusion, this study provides insights into the sequence of accumulation of the key components in the ripening of the bean (Fig. 4). Importantly, the co-expression network illustrated the importance in bean storage and ripening of expansin A transcripts which have a wide interaction with other key components DEGs. This information will facilitate the genetic control of these components in coffee. This ripening bean transcriptome will also provide a platform to determine the genotype and environment influences on coffee quality. Targeted analysis is now possible to characterize gene families of interest (e.g. transcriptional factors). Phylotranscriptomic analysis is another approach now enabled for the study of evolutionary diversity features.

## Methods

### RNA sample and cDNA library preparation

Coffea arabica cv. K7 cherries of different ripening stages (green, yellow and red) were collected from the upper canopy only as described previously^10^. Total RNA isolated from nine samples (three development stages in triplicates) was processed individually according to Furtado *et al*.^39^. The integrity of total RNA was accessed with an Agilent RNA 6000 nano kit and chips through a Bioanalyzer 2100 (Agilent Technologies, California, USA). Thereafter, a standard 18 x Truseq total RNA library preparation was conducted with the use of an additional Ribo-Zero kit. Samples were subsequently sequenced on an Illumina HiSeq4000 platform (2 × 150 bp paired-end reads).

### Reads mining and RNA-Seq analysis

Raw reads were mainly processed with CLC Genomic Workbench 10.0.1 (CLC Bio, Denmark) as following. (1) Adapters and indexes were trimmed. (2) Reads failed matching the PHRED score (<0.01) and length (≥40 bp) were removed. (3) RNA-Seq analysis (read similarity 0.9, length similarity 0.8) was conducted with the processed reads. A recent published long-read sequencing coffee bean transcriptome was used as a reference with Transcripts Per Kilobase Million (TPM) as the expression parameter^3^. Outlier expression values, classified with coefficient variation and standard deviation, were not considered in this study.

### Statistical analysis

Transcripts expressed at each development stage were filtered with TPM (>1). Functional annotations were analyzed with BLAST2GO for GO terms and KEGG pathway distribution^40,41^. Venn diagrams were built through an online tool^42^. Highly expressed transcripts were filtered with TPM (>500). Differential gene expression tool for RNA-seq (CLC) was used for significant through ripening stages. DEGs were filtered with FDR p-value correction (<0.01) and maximum group means (TPM ≥ 10).

Key storage component association with DEGs was constructed through Mercator and Mapman 3.6.0RC1^43,44^. Co-expression network was constructed with Mapman annotated storage DEGs and candidate genes from the targeted analysis. Gene expression of these transcripts were log2(x) transformed before analysis through WGCNA build-in Web MEV package (cutoff_0.9) and Cytoscape 3.5.1.^45,46^ (Note: 0.0001 was assigned to transcripts with an expression value of 0 before log2 transformation).

### Data availability

The RNA-sequencing trimmed data used in this manuscript has been submitted to EMBL database under accession number: PRJEB24137. Please note that the two subsets of the paired-end reads were trimmed together in the data mining process. Two sub-files were generated after trimming, paired reads and orphans. These two sub-files were combined, compressed and submitted to EMBL as “one Fastq file (Single)”.

## Electronic supplementary material

Supplementary information

Supplementary dataset
