# Supplementary material for: The coffee bean transcriptome explains the accumulation of the major bean components through ripening
Source: Sci Rep. 2018 Jul 30;8:11414. doi: 10.1038/s41598-018-29842-4 (PMC6065352; doi:10.1038/s41598-018-29842-4)
Supplement: Supplementary file 1 — Supplementary information [file 41598_2018_29842_MOESM1_ESM.pdf]

The coffee bean transcriptome explains the accumulation of the major bean components through ripening

Bing Cheng, Agnelo Furtado, Robert J. Henry*

Queensland Alliance for Agriculture and Food Innovation, The University of Queensland, Brisbane, QLD 4072, Australia

*Corresponding author: robert.henry@uq.edu.au

Table S1 Overview of RNA-Seq results. Green1, 2, 3, three replicates of Green stage; Yellow1, 2, 3, three replicates of Yellow stage; Red1, 2, 3, three replicates of red stage.

**Sample ID**	**Raw reads**	**Trimmed reads**	**Mapped genes**
Green1	17,355,392	16,980,672	59.04
Green2	30,315,140	29,612,113	52.25
Green3	9,793,436	9,568,758	57.08
Yellow1	8,170,498	7,942,370	56.54
Yellow2	24,656,422	23,905,608	59.9
Yellow3	12,768,562	12,350,938	64.05
Red1	7,642,464	7,405,192	60.19
Red2	4,741,574	4,602,943	58.36
Red3	5,341,298	5,170,766	48.37
Sum	120,784,786	117,539,360	

Table S2 Top 30 KEGG pathways and their corresponding parent groups in ripening coffee bean.

**Ranking**	**KEGG Pathways (TOP 30) expressed ripening coffee seeds**	**Parent-groups of pathways**
1	Purine metabolism	Nucleotide metabolism
2	Thiamine metabolism	Metabolism of cofactors and vitamins
3	Biosynthesis of antibiotics	Global and overview maps
4	Aminobenzoate degradation	Xenobiotics biodegradation and metabolism
5	T cell receptor signalling pathway	Immune system
6	Starch and sucrose metabolism	**Carbohydrate metabolism**
7	Glycolysis/ Gluconeogenesis	**Carbohydrate metabolism**
8	Th1 and Th2 cell differentiation	Immune system
9	Phenylpropanoid biosynthesis	Biosynthesis of other secondary metabolites
10	Pyrimidine metabolism	Nucleotide metabolism
11	Drug metabolism-other enzymes	Xenobiotics biodegradation and metabolism
12	Pyruvate metabolism	Carbohydrate metabolism
13	Amino sugar and nucleotide sugar metabolism	Carbohydrate metabolism
14	Glutathione metabolism	Metabolism of other amino acids
15	Cysteine and methionine metabolism	Amino acid metabolism
16	Glycerolipid metabolism	Lipid metabolism
17	Galactose metabolism	Carbohydrate metabolism
18	Carbon fixation in photosynthetic organisms	Energy metabolism
19	Drug metabolism-cytochrome P450	Xenobiotics biodegradation and metabolism
20	Glyoxylate and dicarboxylate metabolism	Carbohydrate metabolism
21	Methane metabolism	Energy metabolism

22	Pentose phosphate pathway	Carbohydrate metabolism
23	Metabolism of xenobiotics by cytochrome P450	Xenobiotics biodegradation and metabolism
24	Glycine, serine and threonine metabolism	Amino acid metabolism
25	Fatty acid degradation	Lipid metabolism
26	Glycerophospholipid metabolism	Lipid metabolism
27	Valine, leucine and isoleucine degradation	Amino acid metabolism
28	Lysine degradation	Amino acid metabolism
29	Alanine, aspartate and glutamate metabolism	Amino acid metabolism
30	Oxidative phosphorylation	Energy metabolism

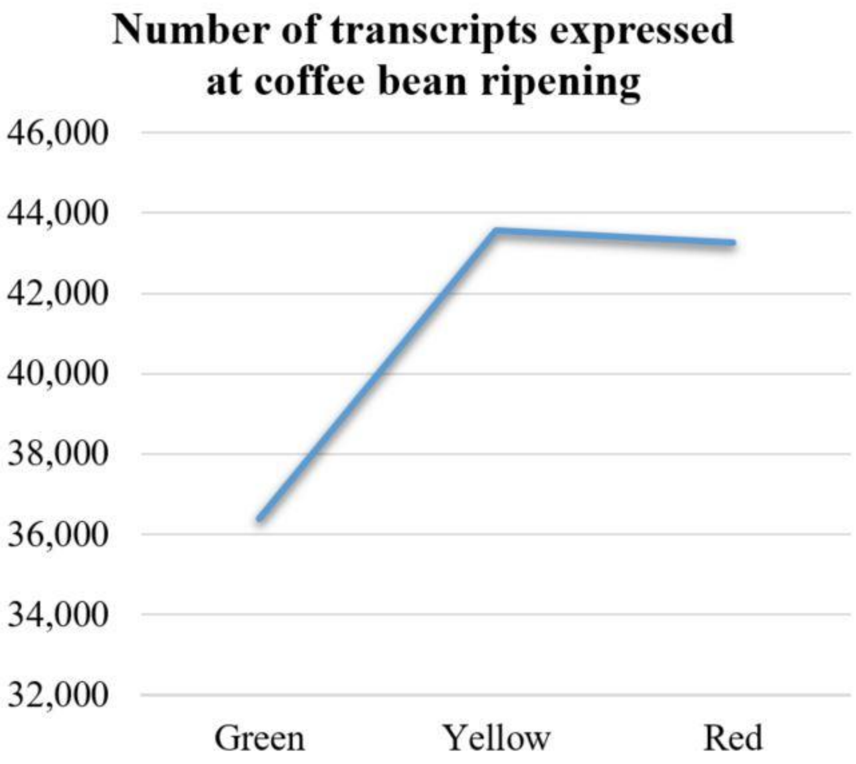

Figure S1 Number of transcripts expressed in green, yellow and red stages of Arabica coffee bean (TPM>1).

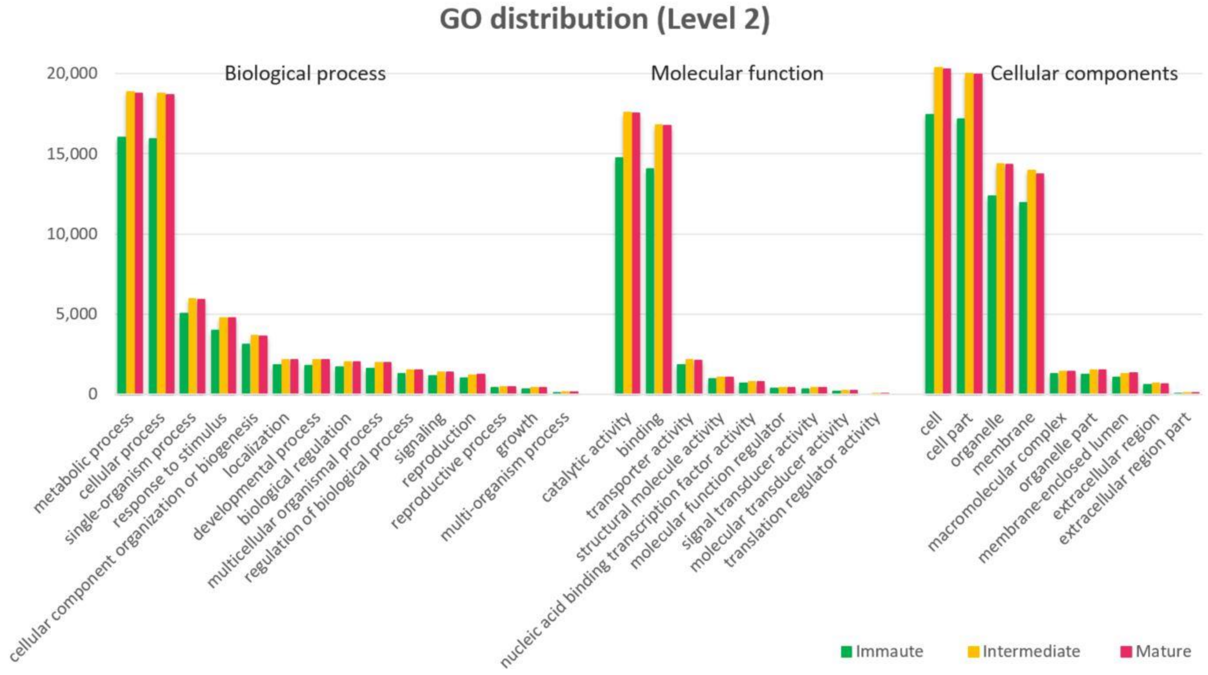

Figure S2 GO distribution in developing coffee bean, including biological process, molecular function and cellular components.
